# Down Regulation of c-FLIP_L_ Enhance PD-1 Blockade Efficacy in B16 Melanoma

**DOI:** 10.3389/fonc.2019.00857

**Published:** 2019-09-04

**Authors:** Yao Wang, Jing-jing Li, Hong-jun Ba, Ke-feng Wang, Xi-zhi Wen, Dan-dan Li, Xiao-feng Zhu, Xiao-shi Zhang

**Affiliations:** ^1^Biotherapy Center, Sun Yat-sen University Cancer Center, Guangzhou, China; ^2^Medical Oncology Department, Affiliated Cancer Hospital & Institute of Guangzhou Medical University, Guangzhou, China; ^3^Pediatric Cardiology Department, Heart Center, The First Affiliated Hospital of Sun Yat-sen University, Guangzhou, China; ^4^Department of Thoracic Surgery, Sun Yat-sen Memorial Hospital of Sun Yat-sen University, Guangzhou, China

**Keywords:** c-FLIP_L_, PD-1, PD-L1, B16 melanoma, immune therapy

## Abstract

Immune checkpoint blockade of programmed cell death protein 1 (PD-1) had an impressive long-lasting effect in a portion of advanced-stage melanoma patients, however, this therapy failed to induce responses in several patients; how to increase the objective response rate is very important. Cellular FLICE-inhibitory protein (c-FLIP) could inhibit apoptosis directly at the death-inducing signaling complex of death receptors and is also considered to be the main cause of immune escape. The overexpression of c-FLIP_L_ occurs frequently in melanoma and its expression is associated with the prognosis. We found that the level of c-FLIP_L_ expression was associated with the PD-1 blockade response rate in melanoma patients. Thus, we performed this research to investigate how c-FLIP_L_ regulates immunotherapy in melanoma. We demonstrate that down regulation of c-FLIP_L_ enhances the PD-1 blockade efficacy in B16 melanoma tumor model. Down regulation of c-FLIP_L_ could increase the tumor apoptosis and enhance the antitumor response of T cells in the lymphocyte tumor cells co-culture system. Moreover, knockdown of c-FLIP_L_ could decrease the expression of PD-L1 and recruit more effector T cells in the tumor microenvironment. Our results may provide a new combined therapeutic target for further improving the efficacy of PD-1 blockade in melanoma.

## Introduction

PD-1 and its ligand PD-L1 signaling pathway are important mechanisms that tumors use to escape antitumor immunity. Recent clinical trials of immune checkpoint blockade displayed obvious long-lasting responses in patients with melanoma, but the effective rate fluctuates between 20 and 30%. In many cases, immunotherapy is ineffective or produces secondary drug resistance (1–4). Thus, it is imperative to develop more aggressive treatment which could improve the efficiency and cure rate of patients.

In order to help understanding the tumor immune response and designing the immunotherapy, Daniel S proposed the concept of “cancer-immunity cycle,” referring to seven key links initiated in the anticancer immune response which could lead to effective killing of cancerous cells (5). In theory, combined treatment, especially with targeted therapy, is necessary to maximize therapeutic benefits (6). Moreover, the therapeutic effect of checkpoint blockade could be further improved by an enhanced killing effect of T cells. There are two dominant apoptosis-related signaling pathways: the Fas/Fas ligand (FasL) mediated ligand-binding external pathway and the mitochondrial dependent internal pathway (7). Fas/FasL pathway, which could mediate “counter-attack,” plays a critical role in maintaining the immune system. As a result, activated tumor-infiltrating lymphocytes can induce apoptosis of tumor cells (8). Thus, more efficient immune therapy depends on the functional integrity of the extrinsic pathway.

Cellular FLICE (FADD-like IL-1β-converting enzyme)-inhibitory protein (c-FLIP) is a key anti-apoptotic regulator that inhibits cell death mediated by the death receptors Fas, DR4, DR5, and TNF-R1 (9). Increased expression of c-FLIP can block the expression of Caspase-8, thus making cells resistant to receptor-mediated apoptosis (10). Thus, elevated expression of c-FLIP is associated with tumor cells escaping from immune surveillance *in vivo*, correlates with a more aggressive tumor, and is also considered to be the main cause of immune escape (11). Previous studies indicate that c-FLIP is associated with chemotherapeutic resistance in many human malignant tumors. In the experimental model, interference with the expression of c-FLIP makes cancer cells more sensitive to chemotherapy and death ligand (10, 12).

It has been found that c-FLIP was overexpressed in numerous solid tumors and hematological neoplasms (9). The expression of c-FLIP was increased in melanoma tissue and its expression was significantly associated with the histological type, Clark Level and Ki-67 labeling index (13). However, the association of c-FLIP expression with the efficacy of PD-1 blockade has not been studied. Therefore, we performed this research to detect the relationship between c-FLIP expression and the efficacy of PD-1 blockade and to further explore the specific mechanisms in the B16 melanoma mouse model.

## Materials and Methods

### Cell Lines, Cell Culture, and Drugs

Human melanoma cell lines MM200, Sk-mel-1, Sk-mel-28, Sk-mel-110, Sk-rm-bcl2, and mouse melanoma cell line B16-F10 were purchased from American Type Cell Culture (Manassas, VA, USA). B16-F10 was cultured in a high-glucose (25 mM) DMEM medium. The other cell lines were cultured in RPMI-1640 medium supplemented with 10% fetal bovine serum, penicillin (50 U/mL), and streptomycin (50 μg/mL). All cells were placed in a humidified incubator at 37°C with 5% carbon dioxide. ERK1/2 inhibitor (SCH772984) and AKT1/2/3inhibitor (MK-2206 2HCL) were purchased from Selleck Chemicals (Houston, TX, USA). PD-1 blockade, Hamster mAb (clone G4C2) against mouse PD-1 was provided by Suzhou Junmeng Bioscience Corporation (Jiang Su, China), which was stored at −20°C.

### Plasmids and Cell Transfection

Short hairpin RNA (shRNA), specific to Mus musculus CASP8 and FADD-like apoptosis regulator (c-FLIP_L_), was designed according to NCBI Reference Sequence: NM_207653.3. Plasmids were constructed by FulenGen Corporation (Guangzhou, China). Lentiviral particles expressing c-FLIP_L_ shRNA and GFP were produced in 293T packaging cells (Invitrogen) using the Lenti-Pac FIV Expression Packaging Kit (GeneCopoeia, USA). Then, B16-F10 cells were infected by retrovirus supernatant in Opti-MEM Medium with the presence of 5 μg/ml Polybrene for 12 h. After infection, stably transduced B16-c-FLIP_L_-shRNA and B16-c-FLIP_L_-control cells were selected using fresh 10% FBS-supplemented medium containing 0.8 μg/ml puromycin for 2 weeks. The transfection efficiencies were determined by fluoroscope for calculating the percent of GFP-expressed cells above 80%.

### Western Blot Analysis

Whole-cell extracts were generated by direct lysis with RIPA lysis buffer (Thermo, Hercules, CA) and Halt Protease and Phosphatase Inhibitor Cocktail (Thermo, Hercules, CA) were added immediately before use. The protein concentration was determined using Thermo Protein Assay Reagent (Thermo, Hercules, CA). Samples were boiled by addition of 5^*^SDS sample buffer for 5 min at 100°C and resolved using 10% SDS-PAGE. Signals were detected by Super Signal West Pico Chemiluminescent Substrate (Thermo, Hercules, CA). Antibodies against GAPDH(2118S), PD-L1(1368s), ERK1/2(4058), phospho-ERK1/2(4370s), phospho-c-Jun(9164), AKT(4685), and phospho-Akt(3787) were purchased from Cell Signal Technology (Danvers, MA, USA); PD-L1/CD274 (mouse) was purchased from Proteintech Group (Chicago, IL, USA); and c-FLIP_L_ and PRAS40 (phosphor T246) were purchased from Abcam (Cambridge, UK). Image acquisition and quantitation of band intensity were performed using ImageJ software (https://imagej.nih.gov/ij/download.html).

### Quantitative Real-Time PCR

Total cellular RNA was isolated with TRIzol reagent (Invitrogen). Samples were treated with DNase using the RNase-free DNase Set (Qiagen) during the total RNA isolation. First-strand complementary DNA (cDNA) was synthesized using the cDNA Synthesis kit (Thermo Fisher Scientific) according to the manufacturer's instructions. ABI prism 7900-HT sequence detection system (96-well, Applied Biosystems) was used to perform quantitative real-time PCR (RT-PCR) analysis. For RT-PCR, the following primers were used:

c-FLIP_L_: 5'-AGAACCTGGCTGCACCTAAC-3'(forward),
5'-GAGAAGGTCAAACCGCCTCA-3'(reverse).
GAPDH: 5'-CGAGATCCCTCCAAAATCAAGTGGGG-3'(forward),
5'-ACACGTTGGCAGTGGGGACAC-3'(reverse).

Each experiment was repeated three times and the relative expression of c-FLIP_L_ was normalized to GAPDH expression.

### Experimentation on Animals

C57BL/6 mice (6- to 8-week-old males) were purchased from Guangdong Medical Lab Animal Center. They were injected subcutaneously with 2 × 10^5^ B16-c-FLIP_L_-shRNA or B16-c-FLIP_L_-control stable cells in the right flank region. Mice received i.p. injections of 100 μg anti-PD-1 antibodies from the 5th day, and additional treatments of 100 μg were given every 3 days until the end of the experiment. Tumor volume was assessed every 3 days from the 5th day. Mice were humanely euthanized on the 16th day or when tumors exceeded 1.8 cm in diameter, and the tumors were dissected and analyzed. All of the disposals were in accordance with the guideline of animal ethics and were approved by the Subcommittee on Research Animal Care of Sun Yat-sen University.

### Isolation of Spleen and Tumor Infiltrating Lymphocytes (TILs)

Spleens were harvested and single-cell suspensions were prepared by processing the spleen with a 200 mm nylon mesh. Then the cell suspension was carefully added to the surface of 5 ml of EZ-Sep Mouse lymphocyte separation medium (Dakewe Biotech Company Ltd., Shenzhen, China) into a 15-ml centrifuge tube. Lymphocytes at the interface were isolated by Ficoll density centrifugation at 800 g for 30 min at 4°C. After red blood cell lysis (Sigma), spleen lymphocytes were collected and washed. Excised tumors were cut into small pieces and digested in RPMI medium containing Liberase at 25 μg/mL (Roche) and DNase1 at 150 UI/mL (Roche) with constant stirring for 30 min at 37°C. The resulting suspension was passed through a 70 μm cell strainer, washed once with HBSS, and then TILs were isolated by Ficoll density centrifugation at 800 g for 30 min at 4°C.

### Flow Cytometry

Staining was performed on splenocytes or monocellular suspensions from mouse tumors. For membrane staining, 1^*^10^6^ splenocytes or TILs were incubated with purified anti-mouse CD3, CD4, CD8, CD25 for 30 min at 4°C. For intracellular staining, the FoxP3 staining kit was used. In a co-cultured system, the apoptosis of CD3-positive T cells and GFP-positive B16 cells were evaluated using an Annexin V-PI apoptosis assay, and staining was performed according to the manufacturer's instructions. Stained samples were detected and analyzed by eight-color flow Cytometry (BD Biosciences, FACS Calibur). Anti-Mouse CD3 (FITC, PC7), CD4 (APC), CD8a (PE), CD25 (PC7), FoxP3 (PE), the FoxP3 staining kit, and the Annexin V Apoptosis Detection Kit were purchased from (eBioscience, USA).

### Immunofluorescence

B16-c-FLIP_L_-shRNA and B16-c-FLIP_L_-control stable cells were grown on glass coverslips. After 12 h of adherent growth, cells were fixed with 4% paraformaldehyde at room temperature for 15 min and washed three more times with phosphate-buffered saline (PBS). Then cells were incubated with primary PD-L1 antibody at 4°C overnight and secondary antibody (Life Technologies, LA) for 1 h at room temperature. After incubating with DAPI (Life technologies) for 5 min, slides were observed and photographed using fluorescence microscopy. The fluorescence intensity were quantitatively analyzed using ImageJ (https://imagej.nih.gov/ij/download.html). These experiments were triplicated repeated.

### Tumor Cells Co-cultured With Splenic Lymphocytes

B16-c-FLIP_L_-shRNA and B16-c-FLIP_L_-control stable cells were seeded into 24-well plates at a density of 3 × 10^4^ cells per well overnight. Splenic lymphocytes were isolated from the spleen of healthy C57BL/6 mice, and then added to 24-well plates. Co-cultured cells were treated with anti-PD-1 antibody (50 ng/ml) or vehicle. After 12 h of treatment, cells were harvested from the co-culture system and stained with anti-mouse CD3 antibody (PC7) for apoptosis assay of lymphocytes or tumor cells. The cell-free supernatant from the co-cultured system was collected for IFN-γ analysis using the mouse IFN-γ ELISA kit (MultiSciences Biotech, Zhejiang, China).

### Cell Viability Analysis

B16-c-FLIP_L_-shRNA and B16-c-FLIP_L_-control stable cells were seeded into 96-well plates at a density of 5 × 10^3^ cells per well and co-cultured with splenic lymphocytes at a lymphocytes/tumor cells ratio of 5:1. After 24 h of treatment, we removed the culture supernatant including suspended lymphocytes and tumor cells, and then washed the attached tumor cells with culture medium three times. Cell viability after treatment was determined using the MTT assay (Toxicology Assay Kit; Sigma-Aldrich, St. Louis, MO, USA). The viability of the vehicle group was normalized to 100%. Cell survival rate (%) = (OD value of treatment group/OD value of vehicle group) × 100%. All experiments were performed in triplicate.

### Immunohistochemistry

Tumors from patients were fixed in 4% paraformaldehyde (PFA), embedded in paraffin, sectioned and stained with haematoxylin and eosin. Immunohistochemical staining was performed using ABC Elite immunoperoxidase kit according to the manufacturer's instructions. The intensity of cytoplasmic staining was scored as negative = 0, weak = 1, moderate = 2, or strong = 3. The percent of positive cells was scored as 0 (≤10%), 1 (10–24%), 2 (25–49%), 3 (50–74%), and 4 (≥75%). The product index was obtained by multiplying the two scores. The median value was used to distinguish the different groups of immunohistochemical variables in the results; the c-FLIP_L_ expression level was considered low when the final score was ≤6 and high when the final score was more than 6.

### Patient Eligibility and Evaluation

We enrolled 26 advanced melanoma patients (age range, 18–75years) treated with anti-PD-1 antibodies at Sun Yat-sen University Cancer Center (SYSUCC) between 1 July 2014 and 30 June 2017. All of the patients were histologically diagnosed and were classified as stage IV according to the 2009 American Joint Committee on Cancer (AJCC) melanoma staging and classification system. This study was approved by the Institutional Review Board of SYSUCC, and written informed consent was obtained from each patient. Baseline characteristics were obtained from the patients' history, including age, gender, performance status (PS), lactic dehydrogenase level (LDH) and pathological subtype. Progression-free survival (PFS) was calculated from the date of diagnosis to first progression, recurrence after response, last follow-up, or death. We evaluated the efficacy of PD-1blockade according to the Response Evaluation Criteria in Solid Tumors (RECIST 1.1) (14).

### Statistical Analysis

All of the statistical analyses were performed using SPSS 17.0 software (IBM, Armonk, NY). Numerical data are presented as the mean ± standard error of the mean (SEM). A standard two-tailed Student's *t*-test and a paired Student's *t*-test were used for comparison of the numerical data, and *P* < 0.05 was considered to be statistically significant.

## Results

### Correlation Between c-FLIP_L_ Expression and Prognosis

**Figure 1:**
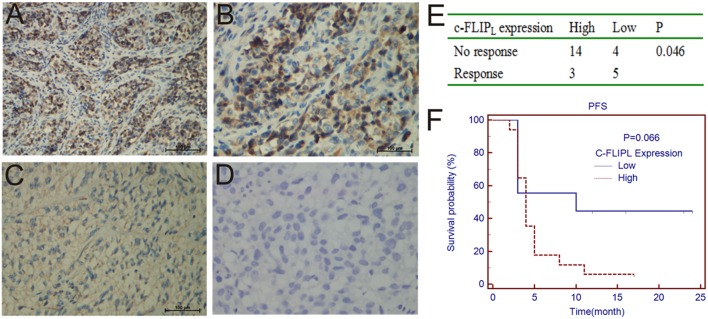
IHC analyses of c-FLIP_L_ expression in melanoma tissues. The representative slides are shown: **(A,B)** are strongly positive, A × 200, B × 400, **(C)** is weakly positive, **(D)** is negative. **(E)**. The c-FLIP_L_ expression vs. response to PD-1 blockade therapy. **(F)**. Progression-free survival in PD-1 blockade treated patients, stratified by c-FLIP_L_ expression.

IHC was performed to determine the correlation between c-FLIP_L_ expression and prognosis. We observed the expression of c-FLIP_L_ in 26 melanoma patients (Figures 1A–D), the characteristic is shown in Table 1. We found that the level of c-FLIP_L_ expression was associated with PD-1 blockade response rate (*P* = 0.046) (Figure 1E). Although the expression of c-FLIP_L_ was not associated with progression-free survival (PFS) (log-rank *P* = 0.066), the c-FLIP_L_ expression seems to trend with the PD-1 blockade response rate (Figure 1F).

**Table 1:** Basic characteristics of 26 melanoma patients with PD-1 blockade therapy.

**Chracteristic**		***N* (%)**
Age	<60	17 (65.4)
	≧60	9 (34.6)
Gender	Male	13 (50)
	Female	13 (50)
PS	0–1	22 (84.6)
	≧2	4 (15.4)
LDH	< ULN	22 (84.6)
	≧ULN	4 (15.4)
Type	Cutaneous	20 (76.9)
	Mucosal	4 (15.4)
	Uveal	2 (7.7)

### Construction of Stable Cell Clones With Different c-FLIP_L_ Expression

**Figure 2:**
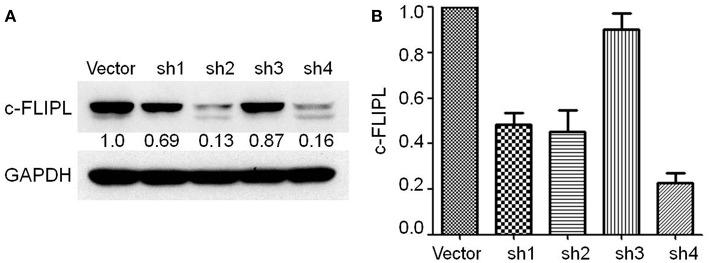
Stable B16-c-FLIP-shRNA and B16-c-FLIP-control cells were generated by transferring a lentivirus-packaged sponge RNA targeting c-FLIP_L_ and control gene. **(A)** c-FLIP_L_ protein expression of different groups and relative quantitative analysis results were labeled under the band. **(B)** mRNA level of c-FLIP_L_ expression in different groups.

We generated B16-c-FLIP_L_-shRNA and B16-c-FLIP_L_-control cells, in which c-FLIP_L_ was knocked down by transferring a lentivirus-packaged sponge RNA targeting c-FLIP_L_ or the control gene. Stable cell clones grown in the presence of puromycin (0.8 μg/ml) were screened out after 2 weeks. The protein and mRNA expression of c-FLIP_L_ was detected by Western blot (Figure 2A) and RT-PCR (Figure 2B). Compared with the negative control group, the protein and mRNA expression of the shRNA4 group was significantly lower than the other three groups. Therefore, c-FLIP_L_ shRNA4 was selected to evaluate the cell function in this study.

### Knockdown of c-FLIP_L_ Enhanced the Efficacy of PD-1 Blockade in B16 Melanoma Tumors

**Figure 3:**
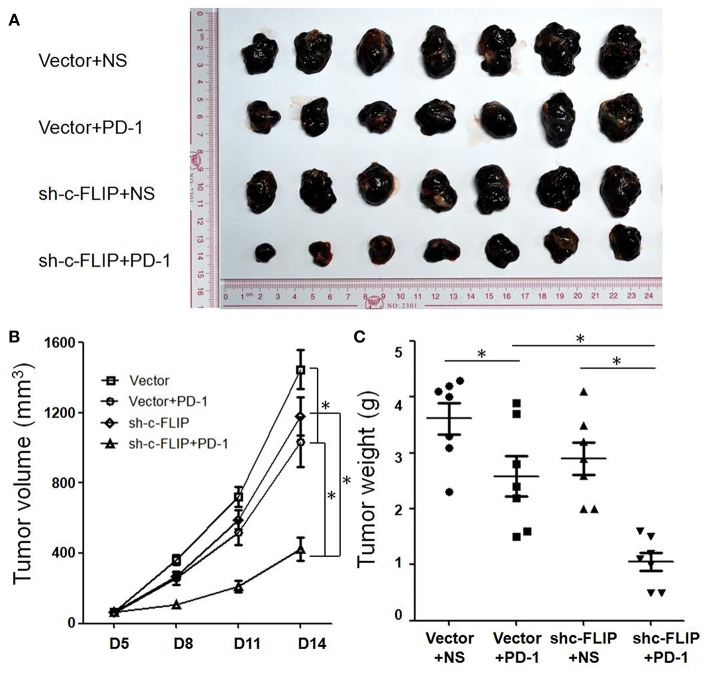
Knockdown of c-FLIP_L_ enhanced the efficacy of PD-1 blockade *in vivo*. **(A)** C57/B6 mice were injected subcutaneously with B16-c-FLIP-shRNA and B16-c-FLIP-control stable cells. After the treatment with PD-1 blockade, tumors were extracted and photographed. **(B)** Tumor growth curves of C57BL/6 mice. Tumor volumes were calculated at the indicated time points. **(C)** Tumor weights on the 14th day. Results are mean volume ± sem for 8 mice per group per time point. ^*^P < 0.05 (Student's *t*-test).

C57/B6 mice were injected subcutaneously with B16-c-FLIP_L_-shRNA and B16-c-FLIP_L_-control stable cells, and then received i.p. injections of 100 μg anti-PD-1 antibodies every 3 days from the 5th day. We found that knockdown of c-FLIP_L_ has tender effect on tumor progression in these animals from the tumor growth curve (Figure 3B). However, these differences were not statistically significant. Treatment with PD-1 antibodies significantly reduced the rate of tumor progression. Strikingly, this treatment in mice bearing B16-c-FLIP_L_-shRNA tumors was more effective (Figures 3A–C), suggesting that knockdown of c-FLIP_L_ significantly augments the efficacy of PD-1 blockade.

### Knockdown of c-FLIP_L_ Increased the Effective T-Cell Ratio Within the Tumor

**Figure 4:**
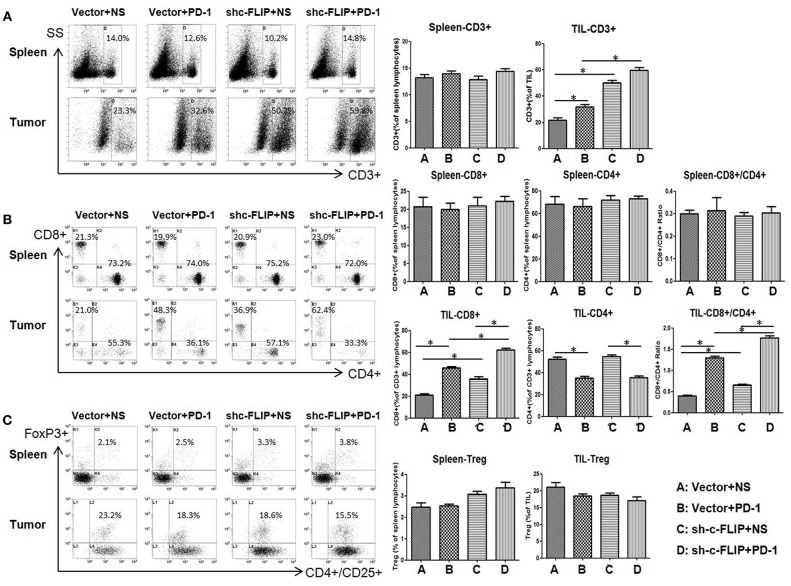
The frequency of T cells in spleen and tumor. **(A)** The frequency of CD3+ T cells could strongly increase in B16-c-FLIP-shRNA tumor groups or with PD-1 blockade. **(B)** The absolute number of CD8+ T-cells and the CD8+/CD4+ Ratio could increase both in B16-c-FLIP-shRNA or PD-1 blockade treated tumor groups. **(C)** The fraction of Tregs in tumor was not diminished in B16-c-FLIP-shRNA tumor groups. ^*^P < 0.05 (Student's *t*-test).

We next investigated the effect of c-FLIP_L_ expression and PD**-**1 blockade on T-cell infiltration in different groups. Contrasting with splenic cells, the frequency of CD3+ T cells strongly increased in B16-c-FLIP_L_-shRNA tumor groups with or without PD-1 blockade (Figure 4A). Downregulation of c-FLIP_L_ and PD-1 therapy alone could increase the CD8+/CD4+ ratio to a similar degree, however, the percentage of CD8+ T cells appear to be increased to a greater extent in B16- c-FLIP_L_-shRNA tumor groups (Figure 4B). PD-1 blockade alone could tenderly decrease the fraction of Tregs in TIL, but the number of Tregs was not diminished with downregulation of c-FLIP_L_ (Figure 4C). From these data, we conclude that knockdown of c-FLIP_L_ could significantly increase the frequency of CD3+ T cells and the percentage of CD8+ T cells more than PD-1 blockade alone.

### Downregulation of c-FLIP_L_ Decreased the Apoptosis Rate of T Cells in a Co-culture System

**Figure 5:**
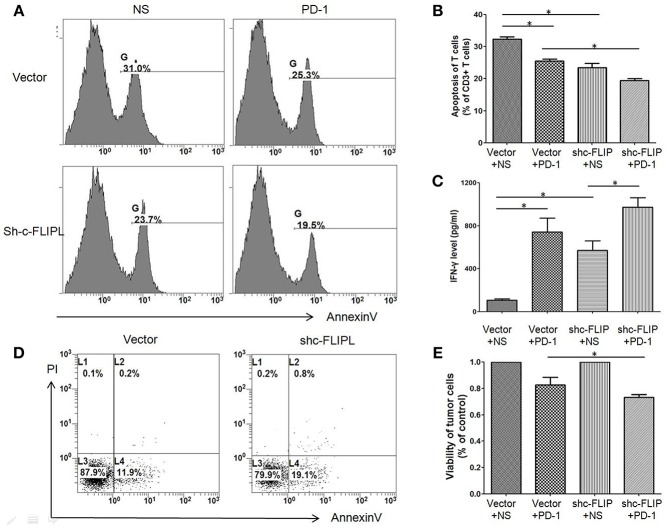
The apoptosis rate of CD3+ T cells and B16 melanoma cells in the co-cultured system. Cells were treated with anti-PD-1 antibody or vehicle for 12 h, and then harvested from the co-culture system and stained for flow cytometry. **(A,B)** The apoptosis rate of CD3+ T cells was higher when co-cultured with B16-c-FLIP-control cells than that with B16-c-FLIP-shRNA. **(C)** IFN-γ was markedly increased in the supernatant of the B16-c-FLIP-shRNA/lymphocytes co-culture system. Downregulation of c-FLIP increased apoptosis rate **(D)** and decreased the viability **(E)** in B16 melanoma cells. These experiments were repeated three times. LC: lymphocyte. ^*^P < 0.05 (Student's *t*-test).

To determine the interaction between lymphocytes and tumor cells, we created a co-culture system in which splenic lymphocytes (From healthy C57BL/6 mice without any treatment) were added to B16-c-FLIPL-control or B16-c-FLIPL-shRNA stable cells at the ratio of 5:1 for 12 h. The apoptosis rate of CD3+T cells was found higher when co-cultured with B16-c-FLIP-control cells than that with B16-c-FLIP-shRNA cells (Figures 5A,B). When supernatants were collected from different co-culture groups, ELISA detection found that the IFN-γ level was markedly increased in the supernatant of the B16-c-FLIP-shRNA/lymphocytes co-culture system (Figure 5C). Moreover, as a key anti-apoptotic regulator, downregulation of c-FLIP_L_ could increase the apoptosis rate (Figure 5D) and decrease the viability (Figure 5E) of B16 cells in a co-culture system. Previous studies showed that PD-L1 in tumor cells can lead to apoptosis or anergy of T cells (15). We found that PD-1 blockade could reduce the apoptosis rate of T cells in both groups (Figure 5A), which indicated that PD-1 blockade may reverse the apoptosis of T cells via the PD-L1/PD-1 pathway. However, it is still unclear whether c-FLIP_L_ could regulate the expression of PD-L1.

### Downregulation of c-FLIP_L_ Could Reduce PD-L1 Expression

**Figure 6:**
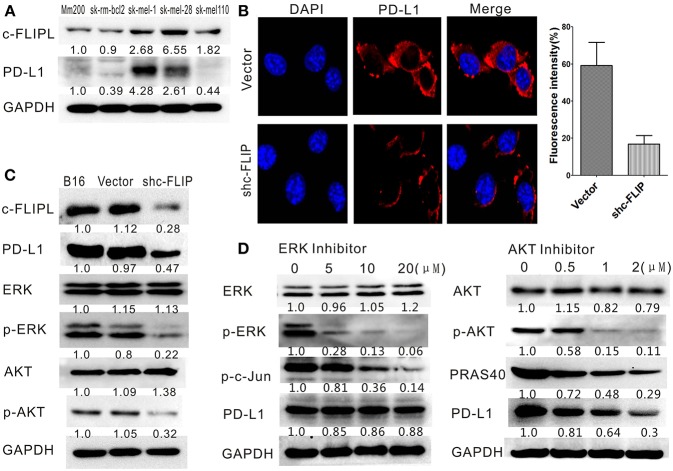
Downregulation of c-FLIP_L_ could reduce PD-L1 expression in B16 melanoma cells. **(A)** The protein level of PD-L1 in melanoma cell lines was in accordance with the protein level of c-FLIP_L_. The protein expression of different groups and relative quantitative analysis results were labeled under the band. **(B)** The different level of PD-L1 expression was detected by immunofluorescence. The fluorescence intensity were quantitatively analyzed. Statistically differences between groups ^*^P < 0.05 (Student's *t*-test). **(C)** Knockdown of c-FLIP_L_ could downregulate the protein expression level of p-ERK, p-AKT, and PD-L1. **(D)** AKT inhibitor effectively suppressed p-AKT and PRAS40 in a dose-dependent manner, which resulted in a decrease of PD-L1 expression.

The protein level of human melanoma cell lines was detected by western blot and we observed the protein level of PD-L1 was in accordance with that of c-FLIP_L_ (Figure 6A). Different levels of PD-L1 expression in B16-c-FLIP_L_-shRNA and B16-c-FLIP_L_-control stable cells were also detected by immunofluorescence (Figure 6B) and Western blot (Figure 6C), which implied that PD-L1 expression could be suppressed by c-FLIP_L_ downregulation. It has been reported that c-FLIP_L_ has multifunctional roles in various pro-survival signaling proteins (10), and we found that knockdown of c-FLIP_L_ could downregulate the protein level of p-ERK and p-AKT (Figure 6C). To further explore which signaling pathway is involved in c-FLIP_L_-induced PD-L1 expression in melanoma, we used ERK or AKT inhibitors to block the pathway in B16-c-FLIP_L_-control cells. We found that the AKT inhibitor (MK-2206 2HCL) could effectively suppress p-AKT and PRAS40 in a dose-dependent manner, which further decreased PD-L1 expression, but the expression of PD-L1 was not altered after using the ERK inhibitor (SCH772984) (Figure 6D). Taken together, our data showed that downregulation of c-FLIP_L_ could reduce PD-L1 expression via the AKT pathway in B16 melanoma cells.

## Discussion

In this research, we explored that the expression of c-FLIP_L_ was correlated with the PD-1 response rate in melanoma patients. Downregulation of c-FLIP_L_ could increase the PD-1 blockade efficacy in the B16 melanoma mouse model, indicating that PD-1 blockade may be more effective in patients with lower c-FLIP_L_ expression, and that c-FLIP_L_ may be the potential target to enhance the efficacy of immune checkpoint blockade.

Recently, predictive biomarkers have been researched and various combination treatments are intensively investigated for enhancing the efficacy of immune checkpoint blockade (16). Studies have shown that the factors predicting successful therapy with PD-1 antibody include the number of infiltrating lymphocytes in the tumor microenvironment, the expression of PD-L1 in the tumor, and the tumor mutation burden (TMB) (17). Many human tumor microenvironments were infiltrated with immune cells. Since the development of cancer is influenced by host immunity, evaluating the number, phenotype and spatial distribution of immune cells in tumors could provide important prognostic information. Recently, the creation of an immunological test called “Immunoscore” was applicable in clinical practice. It is based on quantifying the density of immune cells in the tumor and its invasive margin, particularly total CD3+T-cells and cytotoxic CD8+ T-cells (18). In our experiments, compared with the B16-c-FLIP_L_-control tumor group, we found that the frequency of CD3+ T cells strongly increased in the B16-c-FLIP_L_-shRNA tumor microenvironment. Moreover, the absolute number of CD8+ T-cells and the CD8+/CD4+ ratio increased to a greater extent. This indicates that downregulation of c-FLIP_L_ may alter the tumor microenvironment, and then recruit more T cells.

The effectiveness of PD-1 antibody depends on two important aspects: First, there is a spontaneous anti-tumor immune response in patients. Second, tumor cells could evade immune destruction by expressing PD-L1 (19). PD-L1 expression in tumor cells is the first possible biomarker, and it was reported to be regulated by two main pathways: (1) Adaptive immune tolerance mainly depends on some specific cytokines, such as interferon (IFN)-γ, granulocyte-macrophage colony-stimulating factor (GM-CSF), and interleukin (IL)-4 (20). (2) Inherent immune tolerance depends on the activation of the oncogene signaling pathways, such as PI3K/AKT, the signal transducer and activator of transcription (STAT)-3, epidermal growth factor receptor (EGFR), cyclin-dependent kinase 5 (Cdk5), and MYC-RAS pathways (21–26). The expression of PD-L1, induced by adaptive immune tolerance, represents the intensity of spontaneous immune response, and high expression indicates that the PD-1 antibody is highly effective. However, the PD-L1 expression induced by inherent immune tolerance represents the immune escape ability of the tumor, which is mostly caused by reducing the stability of PD-L1's mRNA. In this case, exogenous PD-1 antibody is not enough to block the interaction between PD-L1/PD-1 molecules, thereby reducing the effectiveness of PD-1 blockade (27). Overexpression of PD-L1 in tumor cells could induce T-cell exhaustion and increase the apoptosis rate of T cells, which was associated with poor prognosis (28). Therefore, specific downregulation of PD-L1 in tumors could improve the efficacy of PD-1 blockade and it is crucial to explore the specific regulation mechanism of PD-L1 expression.

In this report, we found that the protein level of PD-L1 in human melanoma cell lines was in accordance with the protein level of c-FLIP_L_. We also found that downregulation of c-FLIP_L_ could reduce PD-L1 expression in B16 melanoma cells. As we mentioned, PD-L1 could increase the apoptosis of activated tumor antigen-specific T cells. *In vivo* experiment, Dong et al. found that overexpression of PD-L1 on mouse P815 tumor could induce the apoptotic death of activated tumor-reactive T cells. Meanwhile, it can increase the growth rate of tumors with highly immunogenic PD-L1 expression (29). *In vitro* experiment, EGFR activation mediated upregulation of PD-L1 in lung cancer cells could induce the apoptosis of T cells (30). We revealed that inhibiting c-FLIP_L_ could reduce PD-L1 expression, and observed that downregulation of c-FLIP_L_ could decrease the apoptosis of T Cells and increase IFN-γ in the serum of a co-culture system, implying that knockdown of c-FLIP_L_ could reduce the apoptotic death of T cells potentially via the PD-L1/PD-1 pathway. To explore the molecular mechanism of the correlation between c-FLIP_L_ and PD-L1, we tested the downstream pathways of c-FLIP. It was known that c-FLIP_L_ could activate several pro-survival signaling proteins including Akt, ERK, and NF-κB (31). We proved that knockdown of c-FLIP_L_ could downregulate the protein expression level of p-ERK and p-AKT. Moreover, we found that the AKT inhibitor could effectively suppress p-AKT and PRAS40 in a dose-dependent manner, which could further lead to the decrease of PD-L1 expression, but the PD-L1 expression was not changed by using the ERK1/2 inhibitor. Previous studies showed that c-FLIP_L_ is closely related to the interaction with AKT and c-FLIP_L_ enhances the anti-apoptotic functions of AKT by modulating the phosphorylation level of GSK3β (glycogen synthase kinase 3β) (32, 33). Gsk3β could stabilize the expression of PD-L1by regulating the n-glycation modification and induce the phosphorylation-dependent proteasome degradation of PD-L1 (34, 35). All of these studies indicate that c-FLIP_L_ may effect PD-L1 expression through the AKT pathway, but the specific mechanism requires further exploration.

Tumor cells could be depleted by tumor infiltrating lymphocytes through Fas ligand-mediated apoptosis (36). High expression of c-FLIP can block Caspase-8 and makes tumor cells resistant to this apoptotic pathway (37). Therefore, c-FLIP_L_ has been proved to be a key negative regulator of cancer cell apoptosis (38). In line with previous studies, in the lymphocyte-tumor cell co-culture system, we found a higher apoptosis rate in B16-c-FLIP_L_-shRNA cells than in B16-c-FLIP_L_-control cells. We also found the viability of B16-C-FLIP-shRNA cells was much lower than B16-C-FLIP-control cells when co-cultured with splenic lymphocytes, which indicated that downregulation of c-FLIP_L_ could enhance the apoptosis rate of tumor cells.

In summary, our data clarified the relationship between c-FLIP_L_ expression and PD-1 blockade efficacy and highlighted druggable targets to enhance anti-tumor immunity in melanoma. In addition to the pro-apoptosis function, our results showed that downregulation of c-FLIP_L_ could decrease the expression of PD-L1 and enhance antitumor immunity of effector T cells. We found that the level of c-FLIP_L_ expression was correlated with PD-1 blockade response rate in melanoma patients, but the number of patients enrolled in the study is relatively small, and larger sample size would be needed to conclusively determine whether this association is apparently absent in patients. We hope that our study will contribute to the development of new therapeutic targets for combination therapy that could help improve the therapeutic effect of PD-1 blockade.

## Data Availability

The raw data supporting the conclusions of this manuscript will be made available by the authors, without undue reservation, to any qualified researcher.

## Ethics Statement

This study was approved by the Institutional Review Board of SYSUCC, and written informed consent was obtained from each patient.

## Author Contributions

XZha: experimental design and guidance. YW and JL: experiment operation and article writing. HB: animal experiment. KW: streaming data analysis. XW: immunohistochemical analysis. DL: statistic analysis. XZhu: supervising students in the lab.

### Conflict of Interest Statement

The authors declare that the research was conducted in the absence of any commercial or financial relationships that could be construed as a potential conflict of interest.
